# Supplementary material for: Alterations in Bacterial Metabolism Contribute to the Lifespan Extension Exerted by Guarana in Caenorhabditis elegans
Source: Nutrients. 2022 May 9;14(9):1986. doi: 10.3390/nu14091986 (PMC9105138; doi:10.3390/nu14091986)
Supplement: Supplementary file 1 [file nutrients-14-01986-s001.zip › nutrients-1684588-supplementary.pdf]

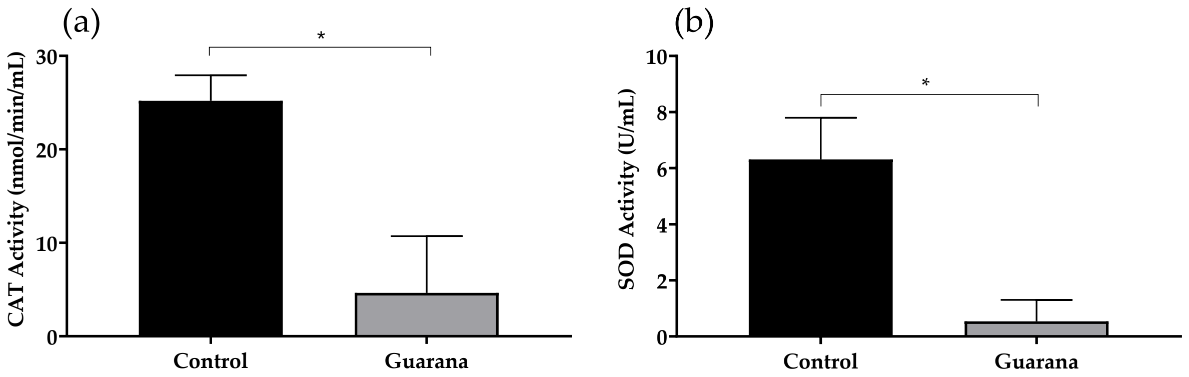

Figure S1. Impact of guarana (100 µg/mL) on the catalase (CAT) (a) and superoxide dismutase (SOD) (b) activity of *C. elegans* (N2). Results are presented as mean and SD of (a) CAT activity (nmol/min/mL) and (b) SOD activity (U/mL). Experiments repeated twice with 300 animals per condition in each experiment (*$p < 0.05$).

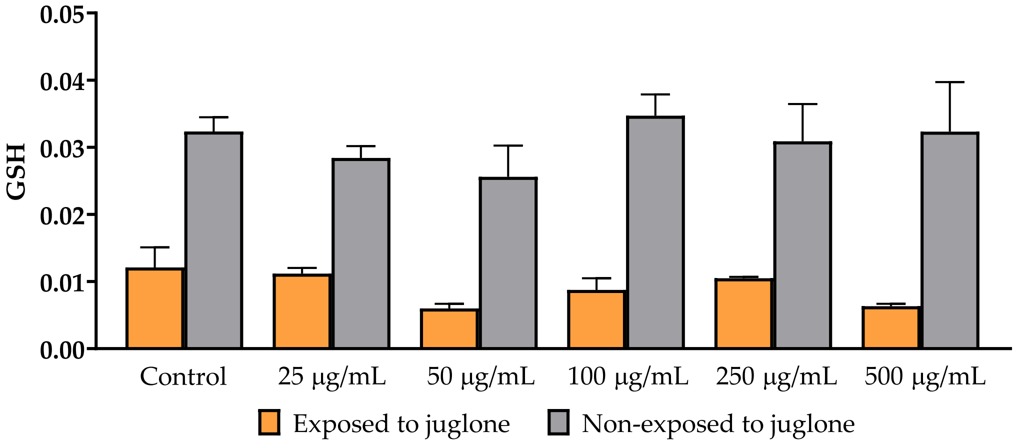

Figure S2. Glutathione (GSH) production of *C. elegans* (N2) when treated with different concentrations of guarana (25, 50, 100, 250 and 500 µg/mL). Orange columns represent GSH production after exposure to oxidative stress caused by juglone, while gray columns represent *C. elegans* GSH production without juglone treatment. Results presented as mean and SEM of GSH levels. Experiments were repeated three times with a total of 150 animals per condition in each experiment.

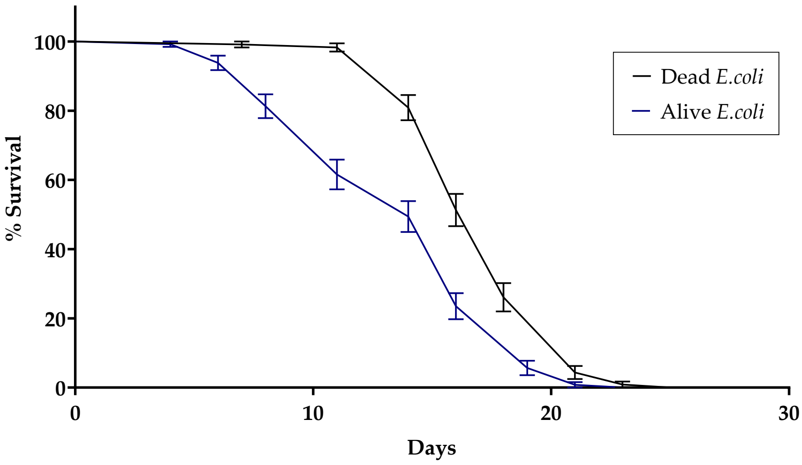

Figure S3. Percentage of survival of SS104 *glp*-4 animals (25°C) fed with alive (n=125) or dead *E. coli* OP 50 (n=115).

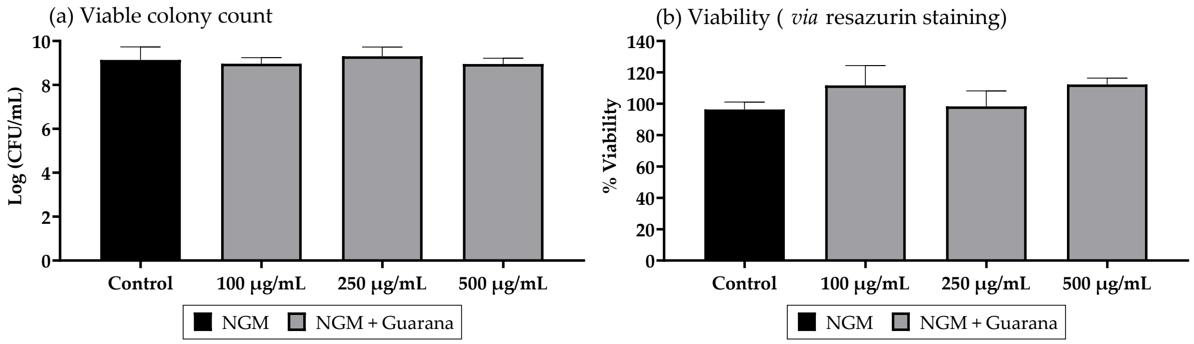

Figure S4. Effects of different concentrations (100, 250 and 500 µg/mL) of guarana on the viable colony counts (a) and the viability (b) of *E. coli.* The effect on viable colony counts (a) is expressed as the log of colony-forming units (CFU)/mL while the viability (b) is expressed as percentage of viable bacterial cells respect the untreated control. Results expressed as mean and SD of three biological repetitions.
